# Changes in Staff Knowledge and Clinical Confidence Following a Structured Teaching Programme on Functional Neurological Disorders: A Two-Cycle Quality Improvement Project

**DOI:** 10.7759/cureus.119372

**Published:** 2026-11-09

**Authors:** Swathi Senthilkumar, Deepanjali Chauhan, Kalpesh Kondalkar, Md Tariqul Islam, Simon Ellis

**Affiliations:** 1 Neurology, Royal Stoke University Hospital, Stoke-on-Trent, GBR; 2 Emergency Medicine, County Hospital, Stafford, GBR; 3 Neurology, University Hospitals Coventry and Warwickshire NHS Trust, Coventry, GBR

**Keywords:** clinical confidence, functional neurological disorder, multidisciplinary education, multidisciplinary teaching, neurological rehabilitation, quality improvement, staff training

## Abstract

Background

Functional neurological disorder (FND) is a common neurological condition requiring accurate diagnosis, effective communication, and coordinated multidisciplinary management. Healthcare professionals’ knowledge and confidence can influence the quality of care provided to patients with FND. This study evaluated baseline staff knowledge, awareness and confidence in managing FND and assessed the impact of a structured multidisciplinary teaching programme on a tertiary neurology ward.

Methods

A questionnaire-based study was conducted among healthcare professionals and support staff involved in patient care on a tertiary neurology ward. Participants included doctors, nurses, healthcare assistants, pharmacy technicians, physiotherapists, occupational therapists and ward clerks. The questionnaire explored knowledge of FND, recognition of positive clinical signs, attitudes towards FND, understanding of treatment options, confidence in clinical management and perception of services in place.

Following the baseline assessment, a dedicated teaching week was delivered, comprising daily sessions on the clinical features of FND, underlying mechanisms, dissociative seizures, nursing management and available treatment resources. A second questionnaire was administered following the intervention to the same set of participants. Responses from the two cycles were compared.

Results

The pre-teaching survey included 45 respondents, and the post-teaching survey included 30. Despite frequent clinical exposure to FND, 73.3% of baseline respondents reported no previous formal FND teaching. Higher self-reported confidence ratings were observed in the post-teaching group across all five assessed domains. The proportion reporting higher confidence (scores of 4-5) in recognising FND was 46.7% before teaching and 86.7% afterwards, while confidence in explaining the diagnosis was 48.9% and 90.0%, respectively. The proportion identifying the complete set of positive clinical signs was 17.8% in the pre-teaching group and 63.3% in the post-teaching group. Recognition that FND can be diagnosed clinically using positive signs was reported by 37.8% and 76.7%, respectively. Recognition of multidisciplinary management was high in both groups (88.9% and 96.7%). These findings represent descriptive differences between anonymous, potentially overlapping respondent groups rather than measured individual improvements.

Conclusion

A dedicated multidisciplinary teaching week was associated with higher self-reported confidence and greater recognition of positive clinical signs of FND among post-teaching respondents. The findings highlight a potential educational gap among staff who frequently encounter FND in clinical practice and suggest that structured, recurring teaching may be beneficial. However, the small sample size, anonymous questionnaires, absence of a control group, and lack of longer-term follow-up or patient outcome measures limit causal interpretation and assessment of sustained benefits.

## Introduction

Functional neurological disorder (FND) is characterised by neurological symptoms arising from altered nervous system functioning and is diagnosed through characteristic clinical features that are ruled in. Presentations include limb weakness, movement disorders, sensory symptoms, and dissociative seizures. FND can cause substantial disability and requires an individualised approach to diagnosis, communication, and treatment [1].

Historically, FND has often been regarded as a diagnosis of exclusion. However, current diagnostic approaches emphasise the identification of positive clinical signs that demonstrate characteristic patterns of inconsistency, rather than relying exclusively on normal investigations [1,2]. Examples include Hoover’s sign in functional limb weakness and entrainment tremor or distractibility in functional movement disorders [3-5]. Familiarity with these signs is therefore very important for healthcare professionals involved regularly in neurological assessment and management.

The management of FND extends beyond just the diagnosis. Physiotherapy, occupational therapy, psychological therapy, and an appropriate multidisciplinary approach are included as part of the treatment, depending on the presenting symptoms and individual needs [6-8]. Clear communication and explanation of the diagnosis are also important in helping patients understand their symptoms and engage with treatment [1,2]. Inadequate knowledge, diagnostic uncertainty and negative attitudes among healthcare professionals may present barriers to appropriate care [9].

Despite increasing recognition of FND, the opportunities for formal education remain limited. Few studies have previously demonstrated that structured multidisciplinary education could improve healthcare professionals’ knowledge and confidence in assessing and managing FND [10]. However, further local quality improvement work is valuable to identify educational needs across different professional groups, particularly among staff who encounter patients with FND during routine inpatient care.

A tertiary neurology ward provides an appropriate setting for such a study because effective management depends on coordinated input from medical, nursing, therapy and support staff. A shared understanding of FND may improve recognition of relevant clinical features, promote consistent communication and support appropriate management of associated symptoms [1,11].

This two-cycle quality improvement study aimed to compare self-reported confidence in five pre-defined domains of FND care amongst multidisciplinary neurology ward staff prior to and following a one-week teaching program, and to compare knowledge, describe attitudes, and identify perceived barriers and educational needs.

## Materials and methods

Study design and setting

This single-centre quality improvement project was conducted at the neurology ward, Royal Stoke University Hospital, Stoke-on-Trent, Staffordshire, United Kingdom, and involved staff participating in the care of patients with FND. Eligible staff included doctors, nurses, healthcare assistants, allied health professionals and other ward-based staff. The project assessed staff knowledge of FND, recognition of positive clinical signs, understanding of treatment approaches and self-reported confidence in clinical management. Participant flow is summarised in Figure 1.

**Figure 1:**
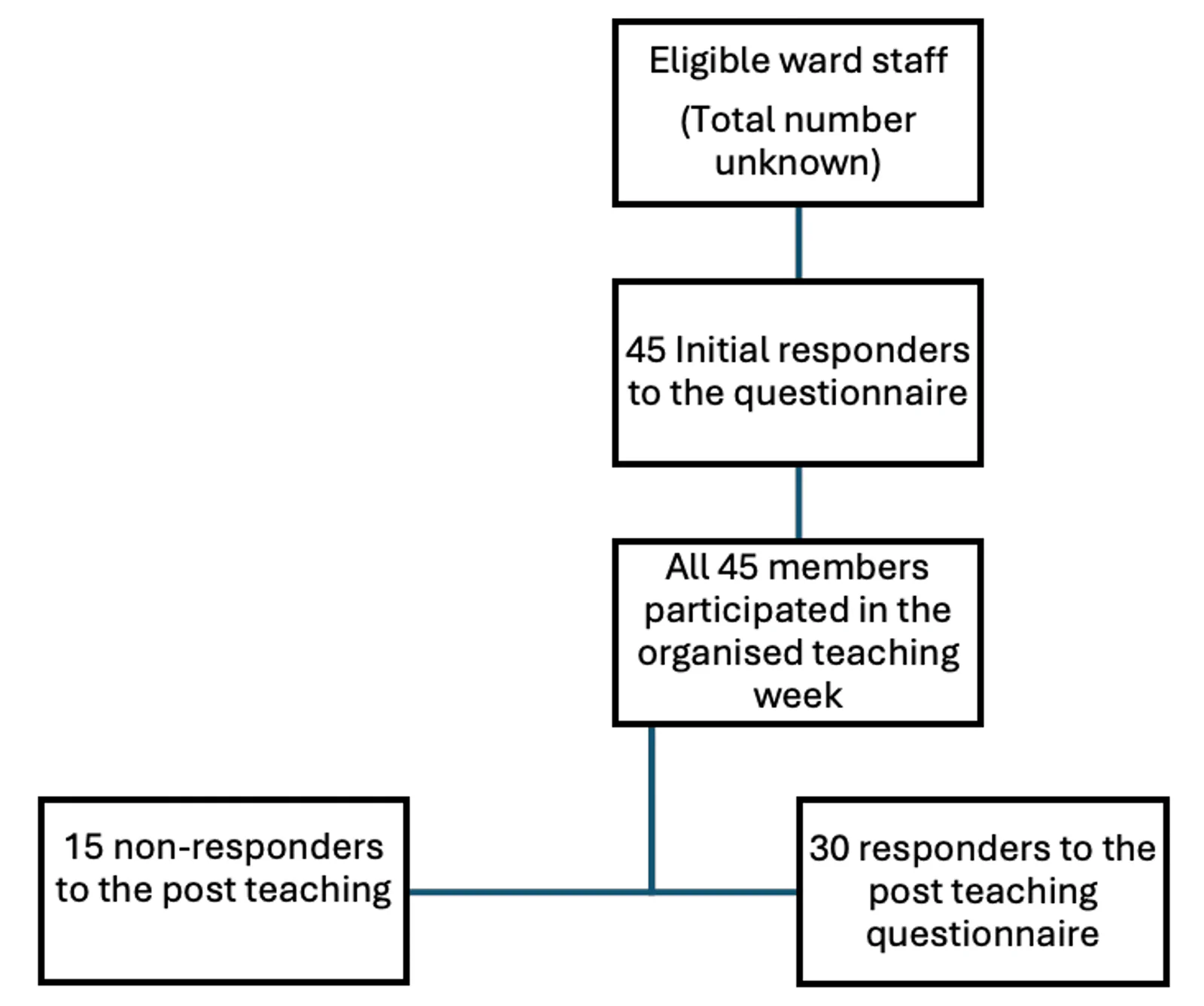
Participant flow diagram

Study process

The pre-teaching questionnaire was administered between the months of May and mid-July 2026, followed by a structured educational programme delivered from 13 to 17 July 2026. The post-teaching questionnaire was administered between 17 July and mid-August 2026. Responses were therefore collected over approximately one month, rather than at a single standardised follow-up time point. No longer-term follow-up assessment was undertaken to evaluate sustained knowledge retention or changes in clinical practice.

Study Tool

The questionnaire (see Appendices) was developed with expert input from a consultant specialising in FND and distributed using a Google Forms link (Google LLC, Mountain View, California, United States), shared by email and QR (quick-response) codes displayed on paper notices. Participation was voluntary, and responses were collected anonymously without names, staff identification numbers, or other identifiers permitting individual responses to be linked.

Both surveys targeted staff working in the same clinical setting. However, individual pre-teaching and post-teaching responses could not be matched, and the extent of respondent overlap was unknown. The total number of eligible staff could not be reliably established; therefore, response rates were not calculated.

The pre- and post-teaching questionnaire comprised 16 numbered questions organised into five sections covering participant characteristics and clinical experience, FND awareness and knowledge, diagnostic and management knowledge, clinical confidence, and perceived service barriers and educational needs. Knowledge was assessed using single-response and multiple-response questions. Attitudes were evaluated using statements concerning the nature, diagnosis, and management of FND. Self-reported clinical confidence was assessed across five domains using a five-point scale ranging from 1 (not confident at all) to 5 (very confident).

Educational Intervention

Following the baseline assessment period, a structured multidisciplinary teaching programme on FND was delivered between 13 and 17 July 2026. The programme comprised six sessions, each lasting approximately 20-40 minutes. Teaching was delivered through lecture-based presentations, case discussions and practical demonstrations. Facilitators included consultants, registrars, junior doctors, occupational therapists and a member of the nursing team with experience and a specialist interest in FND.

The educational content covered clinical presentations and proposed mechanisms of FND: recognition of positive clinical signs, including Hoover's sign, tremor entrainment and symptom distractibility; diagnostic principles and the limitations of relying on normal imaging alone; recognition and management of dissociative seizures; nursing care; multidisciplinary treatment approaches; available clinical resources; and rehabilitation options. The programme concluded with a specialist perspective on FND management. Teaching materials included PowerPoint presentations (Microsoft Corporation, Redmond, Washington, United States), clinical cases, videos and practical demonstrations. Sessions were organised around existing ward activities to facilitate participation by staff working different shifts.

Attendance was not formally recorded. Staff completing the post-teaching questionnaire were instructed to respond only if they had attended the teaching programme. Consequently, individual attendance and the number of sessions attended could not be independently verified.

Study outcomes

The primary outcome was the descriptive difference in FND-related knowledge between the pre-teaching and post-teaching respondent groups. Five selected knowledge outcomes were identified during questionnaire development and assessed using the relevant questionnaire responses. These included recognition of positive diagnostic signs, understanding of clinical diagnosis using positive signs, selection of an appropriate explanation for patients, identification of treatment approaches, and recognition of multidisciplinary management.

The secondary outcome was the descriptive difference in self-reported clinical confidence across five domains: recognising possible FND, explaining the diagnosis, safely managing dissociative seizures, knowing when to refer patients, and distinguishing FND from feigned illness. Attitudes towards FND and perceived barriers to care were also explored descriptively. Differences in question wording and response formats between the questionnaires were considered when interpreting these findings.

Statistical analysis

All available questionnaires were included in the respective descriptive analyses. Data were summarised using frequencies and percentages for categorical variables and means, standard deviations (SDs), medians, and interquartile ranges (IQRs) for self-reported confidence ratings. Higher confidence was defined descriptively as a score of 4 or 5 on the five-point scale.

For selected knowledge outcomes, absolute differences in proportions between the post-teaching and pre-teaching groups were expressed in percentage points. For baseline attitude statements, responses of 1-2 indicated disagreement or strong disagreement, 3 indicated neutrality, and 4-5 indicated agreement or strong agreement. Rejection of negatively worded statements was defined as selecting 1 or 2. 

Because questionnaires were anonymous and individual responses could not be linked, the extent of overlap between respondent groups was unknown. Consequently, paired analysis was not possible, and the independence of the two samples could not be established. No inferential statistical tests were undertaken. Findings were interpreted as descriptive differences between respondent groups rather than individual-level changes attributable to the educational intervention. ChatGPT (OpenAI, San Francisco, California, United States) was used to support statistical data analysis and interpretation.

## Results

Participant characteristics

The initial audit included 45 respondents, while 30 completed the post-teaching questionnaire. Doctors represented the largest professional group in both surveys (51.1% and 56.7%, respectively). Most respondents reported encountering patients with FND or non-epileptic attack disorder (NEAD) weekly (73.3% and 83.3%, respectively). The distribution of clinical experience differed between the groups. Respondents with more than seven years of experience accounted for 33.3% of the initial audit and 13.3% of the post-teaching survey. Participant characteristics are presented in Table 1.

**Table 1:** Comparison of clinical background and experience between participants of both cycles Percentages are calculated using the respective survey sample sizes. FND: functional neurological disorder; NEAD: non-epileptic attack disorder; SHO: senior house officer; FY1: Foundation Year 1

Statement/Question		Initial audit (n=45), n (%)	Post-teaching (n=30), n (%)
Professional role	Doctors (consultants, registrars, SHO/foundation doctors, FY1)	23 (51.1%)	17 (56.7%)
	Nursing staff	10 (22.2%)	4 (13.3%)
	Healthcare assistants	4 (8.9%)	4 (13.3%)
	Allied health/therapy staff	5 (11.1%)	4 (13.3%)
	Other roles or unclear response	3 (6.7%)	1 (3.3%)
Previous formal FND teaching	Yes	12 (26.7%)	17 (56.7%)
	No	33 (73.3%)	13 (43.3%)
Frequency of encountering FND/NEAD	Weekly	33 (73.3%)	25 (83.3%)
	Monthly	8 (17.8%)	4 (13.3%)
	Rarely	1 (2.2%)	1 (3.3%)
	Daily/almost every day	2 (4.4%)	0
	Unsure	1 (2.2%)	0
Experience	Less than 1 year	10 (22.2%)	6 (20.0%)
	1–3 years	9 (20.0%)	10 (33.3%)
	4–7 years	10 (22.2%)	10 (33.3%)
	More than 7 years	15 (33.3%)	4 (13.3%)
	Not reported	1 (2.2%)	0

Despite the high frequency of clinical exposure, only 12/45 (26.7%) respondents in the pre-teaching group reported having previously received formal FND teaching. In the post-teaching group, 17/30 (56.7%) answered yes to this question. This difference should be interpreted cautiously because the post-teaching respondents may have included the educational intervention itself when answering.

Regarding existing services, 31/45 (68.9%) pre-teaching respondents considered FND services at their centre inadequate, nine (20.0%) were uncertain, and five (11.1%) considered them adequate. The post-teaching questionnaire assessed a different aspect of service provision, asking whether respondents felt they could make a difference to FND care. Of the 30 respondents, 28 (93.3%) answered yes and two (6.7%) answered no. These findings describe different perceptions and are not directly comparable measures.

Confidence ratings

Self-reported confidence was assessed across five domains using a five-point scale. Descriptive statistics are presented in Table 2. In the pre-teaching group, mean confidence was highest for knowing when to refer patients for further support (3.67 ± 1.07) and lowest for distinguishing FND from feigned illness (3.07 ± 1.16). In the post-teaching group, mean confidence was highest for recognising possible FND (4.30 ± 0.70), while distinguishing FND from feigned illness remained the lowest-rated domain (3.90 ± 1.21). The proportion of respondents reporting higher confidence (scores of 4 or 5) was greater in the post-teaching group across all five domains. The largest descriptive differences were observed for distinguishing FND from feigned illness (33.3% vs. 76.7%), explaining the diagnosis to patients (48.9% vs. 90.0%) and recognising possible FND (46.7% vs. 86.7%). These findings represent differences between anonymous respondent groups rather than measured changes in individual confidence.

**Table 2:** Descriptive statistics and between initial study and post teaching group SD: standard deviation; IQR: interquartile range; FND: functional neurological disorder

Confidence domain	Pre-teaching (n=45), mean ± SD	Post-teaching (n=30), mean ± SD	Pre-teaching (n=45), median (IQR)	Post-teaching (n=30), median (IQR)	Pre-teaching score 4–5, n(%)	Post-teaching score 4–5, n (%)
Recognising FND	3.38 ± 1.03	4.30 ± 0.70	3 (3–4)	4 (4–5)	21/45 (46.7%)	26/30 (86.7%)
Explaining diagnosis	3.31 ± 1.16	4.27 ± 0.74	3 (2–4)	4 (4–5)	22/45 (48.9%)	27/30 (90.0%)
Safe seizure management	3.53 ± 1.10	4.13 ± 0.94	4 (3–4)	4 (4–5)	27/45 (60.0%)	23/30 (76.7%)
Knowing when to refer	3.67 ± 1.07	4.27 ± 0.78	4 (3–4)	4 (4–5)	27/45 (60.0%)	24/30 (80.0%)
Distinguishing FND from feigned illness	3.07 ± 1.16	3.90 ± 1.21	3 (2–4)	4 (3–5)	15/45 (33.3%)	23/30 (76.7%)

Knowledge outcomes

Knowledge outcomes before and after the teaching intervention are summarised in Table 3. The largest descriptive differences were observed in recognition of positive clinical signs supporting FND diagnosis (17.8% pre-teaching vs. 63.3% post-teaching) and understanding that FND can be diagnosed clinically using positive signs (37.8% vs. 76.7%). For Question 6, four options represented positive clinical signs supporting FND diagnosis: Hoover's sign, tremor entrainment, distractibility, and inconsistency on examination. The option “normal MRI alone” was not considered a positive diagnostic sign. Complete identification was defined as selecting all four appropriate signs without selecting the incorrect option, provided this matched the original scoring criteria.

**Table 3:** Pre-selected knowledge outcomes and comparisons between pre- and post-teaching intervention Percentages are calculated using the respective survey sample sizes.

Knowledge outcome	Initial audit (n=45), n (%)	Post-intervention (n=30), n (%)	Difference (percentage points)
Complete set of positive clinical signs	8 (17.8)	19 (63.3)	45.6
Clinical diagnosis using positive signs	17 (37.8)	23 (76.7)	38.9
Selected “Your symptoms are real”	18 (40.0)	20 (66.7)	26.7
Complete listed treatment set	25 (55.6)	23 (76.7)	21.1
Multidisciplinary management	40 (88.9)	29 (96.7)	7.8

Higher proportions of post-teaching respondents also selected the statement “Your symptoms are real” when explaining FND to patients (40.0% vs. 66.7%) and identified the complete set of appropriate treatment approaches (55.6% vs. 76.7%). Recognition of multidisciplinary management was already high before teaching (88.9%) and remained high afterwards (96.7%).

Overall, the post-teaching group demonstrated higher proportions of responses meeting the specified knowledge criteria, particularly regarding positive diagnostic signs. However, these findings represent differences between anonymous respondent groups rather than directly measured changes in individual knowledge.

Attitudes towards FND

Responses to statements about the nature of FND identified differences in the views expressed by the two respondent groups. In the pre-teaching group, 37/45 (82.2%) agreed or strongly agreed that FND is a genuine neurological disorder. Twenty-six respondents (57.8%) disagreed or strongly disagreed that symptoms are intentionally produced by patients, while nine (20.0%) agreed or strongly agreed with this misconception and 10 (22.2%) selected a neutral response. 

Similarly, 19/45 (42.2%) pre-teaching respondents disagreed or strongly disagreed that FND is purely a psychiatric illness. Twelve respondents (26.7%) agreed or strongly agreed with this statement, while 14 (31.1%) remained neutral. These findings indicate that uncertainty and misconceptions concerning the nature of FND were present in the baseline sample.

In the post-teaching questionnaire, 28/30 (93.3%) respondents were classified as recognising FND as a genuine neurological disorder. Twenty-seven (90.0%) rejected the statement that patients intentionally produce or make up their symptoms, while 26 (86.7%) rejected the description of FND as purely psychiatric. However, the post-teaching questionnaire used a different response format, so comparisons of agreement and disagreement categories should be interpreted cautiously.

The baseline questionnaire also explored perceived barriers to effective FND care. Responses identified staff education, access to psychology or neuropsychiatry services, diagnostic uncertainty, time constraints, stigma, physiotherapy resources, and follow-up pathways as areas of concern. 

Free-text comments provided additional context. Pre-teaching respondents requested more regular education, improved access to specialist psychological and neuropsychiatric services, clearer referral pathways, and greater availability of multidisciplinary rehabilitation. Post-teaching respondents suggested extending FND education to other wards and departments, developing dedicated multidisciplinary services, improving patient education and increasing access to specialist resources.

Taken together, these responses indicate that staff identified educational needs alongside wider organisational and service-related challenges. Although post-teaching respondents expressed confidence in their potential contribution to FND care, the qualitative comments suggest that staff education alone may not address all perceived barriers to effective management.

## Discussion

This two-cycle study identified substantial gaps in formal FND education among multidisciplinary staff on a tertiary neurology ward, despite frequent exposure to patients with the condition. Following a dedicated teaching week, the post-intervention questionnaire demonstrated improvements across the assessed domains of diagnostic knowledge, treatment awareness, and clinical confidence.

The improvement in recognition that positive clinical signs like Hoover’s sign and tremor entrainment are particularly relevant to diagnosing FND, rather than it being a diagnosis of exclusion. Positive clinical signs are central to contemporary FND diagnosis because they can demonstrate characteristic functional abnormalities and help clinicians explain the basis of the diagnosis to patients [1-3]. The observed reduction in respondents selecting normal MRI findings alone as a diagnostic sign also suggests improved understanding that FND should be identified through appropriate clinical features rather than solely through the absence of structural abnormalities found on investigation [1,2].

These findings highlight the importance of teaching diagnostic principles to the whole multidisciplinary team, rather than limiting education to clinicians responsible for making the diagnosis. Nursing and therapy staff frequently support patients during symptom episodes and rehabilitation, and a shared understanding may help promote consistent explanations and care.

Recognition of multidisciplinary management was already high before teaching (88.9%) and remained high afterwards (96.7%). Although a greater proportion of post-teaching respondents met the reported criterion for identifying appropriate treatment approaches (55.6% versus 76.7%), these descriptive differences do not establish that the intervention improved understanding of rehabilitation or altered clinical management. This is an important factor in terms of FND management and has previously published recommendations emphasising the role of physiotherapy and occupational therapy in functional motor symptoms and the importance of individualised, coordinated treatment [6-8]. Education that includes practical management approaches may help staff understand how their respective roles contribute to patient recovery and functional improvement.

Dissociative seizures also require a coordinated and informed response. Clinical management should be based on an appropriate assessment, clear communication, and an individualised management plan. Psychological treatment may be appropriate for selected patients, although treatment outcomes vary and should not be assumed to be uniform [12]. The observed improvement in confidence in managing dissociative seizures is encouraging, but the remaining gap suggests that further practical education may be beneficial.

The findings support previous work demonstrating that structured education can improve healthcare professionals’ knowledge and confidence in FND [10]. A dedicated teaching week offers a practical means of delivering consistent information across professional groups and addressing misconceptions identified through baseline assessment. The importance of such education extends beyond factual knowledge. FND has historically been associated with stigma, diagnostic uncertainty and negative clinical attitudes, which can affect patient experience and access to appropriate care [9,13]. Educational programmes that combine clinical knowledge with communication skills and multidisciplinary discussion may help address these challenges [10].

Recurring teaching, induction resources and case-based discussions could help reinforce the learning achieved during a dedicated teaching week. Future programmes may also benefit from incorporating simulated clinical scenarios, practical demonstrations of positive signs, and structured discussion of dissociative seizure management.

The proportion of respondents reporting higher confidence in explaining FND increased from 48.9% to 93.3%. This represents a substantial difference between the two respondent groups and suggests that the intervention may have improved staff preparedness for discussing the condition. Confidence in safely managing dissociative seizures increased from 60.0% to 76.7%, indicating a more modest improvement. This difference may reflect the practical complexity of seizure management and the need for staff to feel confident in responding to acute episodes. Self-reported confidence should not be equated with demonstrated clinical competence. Nevertheless, it provides a useful educational outcome when interpreted alongside knowledge-based measures and supplemented by objective assessments in future cycles.

Limitations

Several limitations should be considered when interpreting these findings.

First, the project was conducted in a single tertiary neurology ward with a relatively small number of respondents, limiting the generalisability of the findings to other clinical settings. The baseline and post-teaching questionnaires included 45 and 30 responses, respectively. As the questionnaires were anonymous and could not be linked, the extent of participant overlap was unknown. The smaller post-teaching sample may reflect differences in staff availability, engagement, or willingness to participate. Furthermore, the proportion of respondents with more than seven years of clinical experience was lower in the post-teaching group (13.3%) than at baseline (33.3%), suggesting possible differences in sample composition. These factors may have introduced selection bias and prevented assessment of individual-level changes.

Second, the absence of a control group limits the ability to attribute observed differences to the educational intervention. The teaching content also overlapped with several questionnaire items, particularly those assessing positive diagnostic signs. Consequently, familiarity with the assessed material may have influenced post-teaching responses. The post-teaching questionnaire was administered over a limited follow-up period, and no standardised longer-term assessment was undertaken to establish sustained knowledge retention.

Third, the questionnaire was developed for local quality improvement. Its measurement properties were not established. Several items also had design limitations. Question 8 required respondents to select only one statement despite including multiple clinically appropriate explanations of FND, restricting its interpretation as a knowledge measure. Selected attitude statements and response formats differed between the pre-teaching and post-teaching questionnaires, limiting direct comparison. Additionally, the question regarding previous formal FND teaching may have been interpreted differently after the intervention, as respondents could have included the programme itself in their answers.

Fourth, confidence ratings were self-reported and may have been influenced by social desirability bias, particularly when respondents were aware of the educational programme. Increased confidence does not necessarily indicate improved clinical competence. The project did not assess observed clinical behaviour, diagnostic accuracy, referral practices, or patient outcomes.

Finally, because individual responses could not be linked and the degree of overlap between survey groups was unknown, confirmatory inferential comparisons were not undertaken in the revised analysis. The findings were therefore interpreted descriptively and cannot establish individual improvements or a causal relationship between teaching and the observed differences.

## Conclusions

This single-centre quality improvement project identified higher self-reported confidence ratings and greater recognition of selected FND diagnostic principles among staff responding after a structured teaching programme. The largest descriptive differences concerned recognition of positive clinical signs and confidence in explaining and identifying FND. However, the anonymous, unmatched questionnaire design, differences in respondent composition, absence of a control group, and lack of long-term follow-up limit the interpretation of these findings. The results cannot establish individual learning, sustained knowledge retention, or improvements in clinical practice.

Structured multidisciplinary education may be a useful component of local efforts to address FND-related knowledge gaps. Further quality improvement cycles incorporating consistent questionnaires, identifiable but confidential response linkage, attendance monitoring, and longer-term evaluation would help determine whether educational interventions translate into sustained improvements in staff knowledge and patient care.

## Appendices

Study questionnaire

**Table 4:** Staff Questionnaire on Functional Neurological Disorder (FND) Credit: Swathi Senthilkumar, Kalpesh Kondalkar, Simon Ellis

Instructions and Confidentiality
This questionnaire aims to assess awareness, understanding and confidence regarding the diagnosis and management of Functional Neurological Disorder (FND) among healthcare professionals working in the neurology ward. Responses are anonymous and will be used for quality improvement and educational purposes only. Estimated completion time is 7–10 minutes.

Section 1: Demographics and Baseline Experience										
Question	Response options									
1.What is your role?	Consultant				Registrar			SHO/Foundation doctor		
	Nurse				Psychologist/psychiatrist			Occupational therapist		
	Physiotherapist				Healthcare assistant			Speech and language therapist		
	Other									
2.Years of clinical experience?	Less than a year		1–3 years			4–7 years			More than 7 years	
3.Have you previously received formal teaching on FND?	Yes					No				
4.Approximately how often do you encounter patients with FND/NEAD?	Daily/almost everyday	Weekly		Monthly			Rarely			Unsure

Section 2: Awareness and Knowledge Scales		
No.	Statement	Response scale
5.1	FND is a genuine neurological disorder	1 to 5 Likertª
5.2	FND symptoms are intentionally produced by patients	1 to 5 Likertª
5.3	FND can be diagnosed using positive clinical signs	1 to 5 Likertª
5.4	Normal investigations do not exclude significant disability in FND	1 to 5 Likertª
5.5	Patients with FND often experience substantial impairment in quality of life	1 to 5 Likertª
5.6	FND is purely a psychiatric illness	1 to 5 Likertª
5.7	Psychological stressors may contribute to FND in some	1 to 5 Likertª
5.8	FND can coexist with other neurological disorders such as epilepsy, stroke or multiple sclerosis	1 to 5 Likertª
5.9	Dissociative/non-epileptic seizures are a subtype of FND	1 to 5 Likertª
5.10	Early and clear explanation of diagnosis may improve outcomes in	1 to 5 Likertª
Note. ª Response options: 1 = Strongly disagree, 2 = Disagree, 3 = Neutral, 4 = Agree, 5 = Strongly agree.		

Section 3: Knowledge Regarding Diagnosis and Treatment								
Question	Response options							
6.Which of the following supports a positive diagnosis of FND? (Check all that apply)	Hoover’s sign			Entrainment tremor			Distractibility of symptoms	
	Normal MRI alone			Inconsistency on examination			Not sure	
7.Which statement regarding investigations in FND is most accurate? (Mark only one)	Extensive normal investigations are required before diagnosing FND							
	FND is diagnosed only after excluding every organic disorder							
	FND may be diagnosed clinically using positive signs							
	FND cannot coexist with organic neurological disease							
8.Which of the following are appropriate when explaining FND to patients? (Mark only one)	“Your symptoms are real.”		“Nothing is wrong.”			“The nervous system is not functioning properly.”		
	“You are imagining the symptoms.”		“These symptoms can improve with treatment.”					
9.Which treatments may play a role in FND management? (Check all that apply)	Physiotherapy		Psychological therapy			Occupational therapy		
	Education regarding diagnosis		Multidisciplinary rehabilitation			Anti-epileptic drugs for all patients with dissociative seizures		
10.Which statement is most accurate? (Mark only one)	There are no effective treatments for FND				FND management is usually multidisciplinary			
	Only psychiatrists should manage FND				Physiotherapy has no role in FND			
11.In your opinion, which specialties should ideally be involved in FND care? (Check all that apply)	Neurology	Psychiatry			Psychology			Physiotherapy
	Occupational therapy	Speech and language therapy			Pain management			Primary care

Section 4: Confidence and Attitudes		
No.	Statement	Response scale
12.1	Confidence in recognising possible FND	1 to 5 Ratingᵇ
12.2	Confidence in explaining FND diagnosis to patients	1 to 5 Ratingᵇ
12.3	Confidence in managing dissociative/non-epileptic seizures safely	1 to 5 Ratingᵇ
12.4	Confidence in knowing when to refer patients for further support	1 to 5 Ratingᵇ
12.5	Confidence in distinguishing FND from feigned illness	1 to 5 Ratingᵇ
Note. ᵇ Response options evaluated on a 5-point scale: 1 = Not confident at all, 2, 3, 4, 5 = Very confident.		

Section 5: Current Service and Educational Needs						
Question	Response options					
13.Do you think current FND services at your centre are adequate? (Mark only one)	Yes		No		Maybe	
14.What do you think are the main barriers to good FND care? (Check all that apply)	Lack of staff education	Limited psychology/ neuropsychiatry access		Diagnostic uncertainty		Time constraints
	Stigma regarding FND	Lack of physiotherapy resources		Poor follow-up pathways		Other
15.What additional support or education would help you manage FND patients better?	Free Text					
16.Additional comments or suggestions regarding FND care in your institution:	Free Text					
